# Blinding Orbital Apex Syndrome due to Onodi Cell Mucocele

**DOI:** 10.1155/2014/453789

**Published:** 2014-05-15

**Authors:** Efrat Fleissig, Oriel Spierer, Ilan Koren, Igal Leibovitch

**Affiliations:** ^1^Oculoplastic and Orbital Institute, Tel Aviv Sourasky Medical Center, Sackler Faculty of Medicine, Tel Aviv University, 64239 Tel Aviv, Israel; ^2^Department of Ophthalmology, Tel Aviv Sourasky Medical Center, 64239 Tel Aviv, Israel; ^3^Department of Otolaryngology, Tel Aviv Sourasky Medical Center, Sackler Faculty of Medicine, Tel Aviv University, 64239 Tel Aviv, Israel

## Abstract

The onodi cell is a posterior ethmoidal cell that is pneumatized laterally or superiorly to the sphenoid sinus with close proximity to the optic nerve. A mucocele, a benign, expansile, cyst-like lesion of the paranasal sinuses, may uncommonly involve the onodi cell causing compression of the optic nerve and nearby structures. In this paper, we report a rare case of onodi cell mucocele causing orbital apex syndrome, with prompt recovery after endoscopic removal. However, optic neuropathy did not improve and the patient remained blind.

## 1. Introduction

Mucoceles arising from the sphenoid sinus or a posterior ethmoidal cell are uncommon and may result in compression of the optic nerve and adjacent structures, leading to orbital apex syndrome. Onodi cell is a posteriorly positioned ethmoid cell that pneumatizes laterally or above the sphenoid sinus, adjacent to the course of the optic nerve. Mucocele formation in such a cell may lead to compressive optic neuropathy and blindness, as in the case presented here.

## 2. Case Report

**Figure 1:**
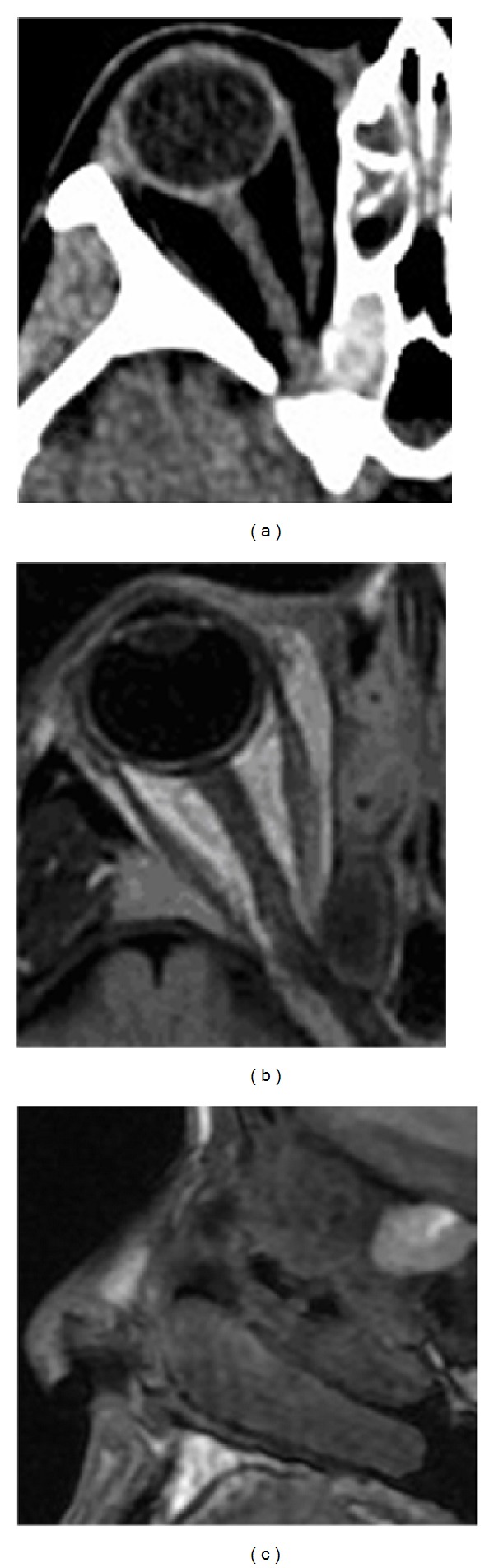
(a) Axial CT showing the mucocele of the right onodi cell. (b) + (c) Fluid attenuated inversion recovery (FLAIR) axial MR image (b) and T2 weighted sagittal MR image. (c) Demonstrating onodi mucocele with close proximity to the optic nerve.

A 53-year-old female presented with vision loss in her right eye that started one day earlier, accompanied by pain in eye movement. On examination, visual acuity was finger counting in her right eye and 20/20 in her left eye. There were also swelling and erythema of the right eyelids and a right relative afferent pupillary defect. The rest of the ophthalmic examination was normal. She was afebrile with no signs of active infection. CT demonstrated opacification of the ethmoidal and sphenoidal air cells parallel to the optic nerve (Figure 1). Patient history was positive for chronic rhinosinusitis that was treated one year earlier with an uneventful bilateral total functional endoscopic sinus surgery (FESS) that included the sphenoid sinuses. With a diagnosis of an inflammatory process, treatment with intravenous methylprednisolone (1 mg/kg per day) was started.

**Figure 2:**
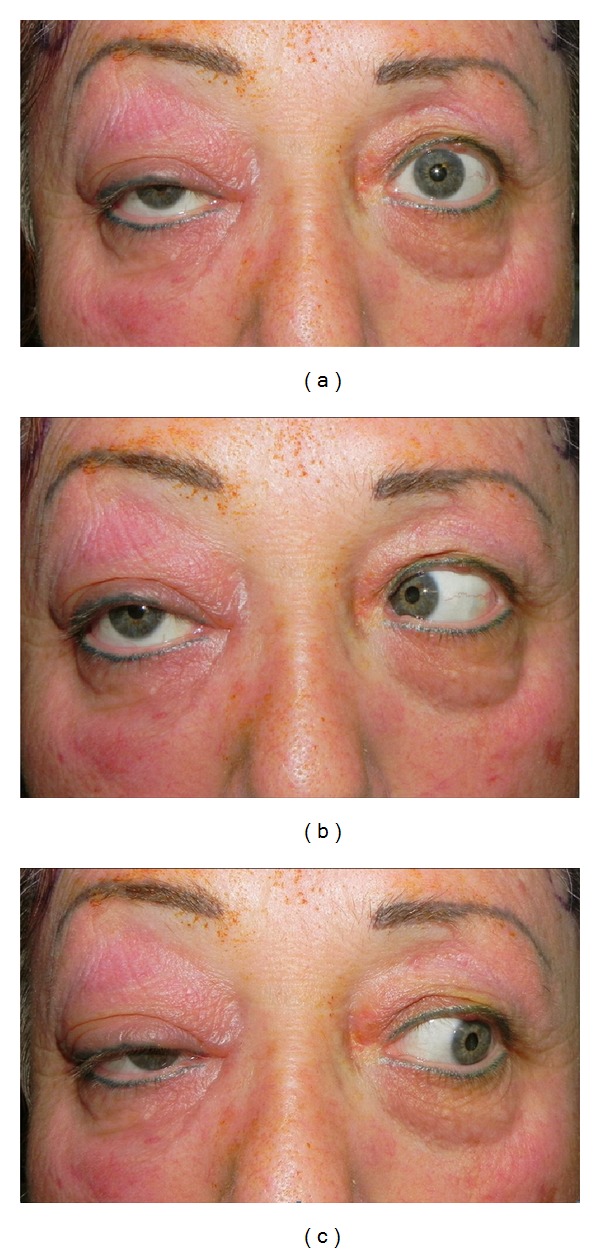
Clinical photograph showing right eye limitation of abduction and adduction.

A few hours later, the patient complained of binocular diplopia and paresthesias along the distribution of cranial nerves V1 and V2. In addition, eyelid swelling had worsened and visual acuity deteriorated to light perception only. An almost complete limitation of abduction and adduction in her right eye was noted (Figure 2). An urgent MRI demonstrated an onodi cell eroding through the lamina papyracea, extending to the superior orbital fissure and compressing the optic nerve, the first 2 divisions of the trigeminal nerve, and the abducens nerve. On the same day the patient underwent an endoscopic sinus surgery with cyst marsupialization and aspiration of a clear mucoid substance. Cultures were not taken due to the clinical observation of a mucocele. During the following days eye movements returned to normal but right optic disc atrophy evolved with visual acuity of no light perception.

## 3. Discussion

There are only a few reports on onodi cell mucocele causing orbital apex syndrome [1–4].

Paranasal sinus mucoceles affect the optic nerve by either mechanical compression causing ischemic injury or by direct extension of the inflammatory process to the optic nerve [5]. It has been suggested that cases with gradual visual acuity decrease are caused by circulatory disorders due to direct pressure of the mucocele, whereas cases that show rapid loss of visual acuity are caused by direct spread of infection or inflammation to the optic nerve [5].

Our patient demonstrated rapid visual loss. When inflammatory signs subsided and no recovery of the optic nerve function was noted, it was suspected that ischemia of the posterior optic nerve may have occurred. The finding of clear mucoid fluid also supports the diagnosis of ischemic injury to the optic nerve. However, considering the acute inflammatory signs, inflammation may also have a role in the presence of optic neuropathy.

Treatment of mucocele is based on prompt endoscopic surgical decompression and systemic steroids to reduce inflammation [1]. When treated early, visual outcome may improve dramatically [1, 2]. Unfortunately the patient's poor prognostic factors, such as sudden onset and poor visual acuity on presentation, led to development of optic atrophy and loss of vision.

In conclusion, clinical signs of orbital apex syndrome in patients with a history of chronic sinusitis should raise the suspicion of mucocele arising from the posterior paranasal sinuses. An immediate orbital and sinuses imaging is required with early surgical intervention. With prompt treatment, vision and eye movements can be restored even though permanent visual loss may occur.
